# Sprint interval training (SIT) reduces serum epidermal growth factor (EGF), but not other inflammatory cytokines in trained older men

**DOI:** 10.1007/s00421-021-04635-2

**Published:** 2021-03-16

**Authors:** Zerbu Yasar, Bradley T. Elliott, Yvoni Kyriakidou, Chiazor T. Nwokoma, Ruth D. Postlethwaite, Christopher J. Gaffney, Susan Dewhurst, Lawrence D. Hayes

**Affiliations:** 1 Active Ageing Research Group, Institute of Health, University of Cumbria, Lancaster, UK; 2 Translational Physiology Research Group, School of Life Sciences, College of Liberal Arts and Sciences, University of Westminster, 115 New Cavendish St, London, W1W 6UW UK; 3 Faculty of Health and Life Sciences, Coventry University, Coventry, UK; 4 Lancaster Medical School, Faculty of Health and Medicine, Lancaster University, Lancaster, UK; 5 Department of Rehabilitation and Sport Sciences, Bournemouth University, Bournemouth, UK; 6 School of Health and Life Sciences, University of the West of Scotland, Glasgow, UK

**Keywords:** Ageing, Cytokines, Exercise, Growth factors, HIIT, Inflammation

## Abstract

**Purpose:**

The present study aimed to investigate the effect of age on circulating pro- and anti-inflammatory cytokines and growth factors. A secondary aim was to investigate whether a novel sprint interval training (SIT) intervention (3 × 20 s ‘all out’ static sprints, twice a week for 8 weeks) would affect inflammatory markers in older men.

**Methods:**

Nine older men [68 (1) years] and eleven younger men [28 (2) years] comprised the younger group. Aerobic fitness and inflammatory markers were taken at baseline for both groups and following the SIT intervention for the older group.

**Results:**

Interleukin (IL)-8, vascular endothelial growth factor (VEGF), and monocyte chemoattractant protein-1 (MCP-1) were unchanged for the older and younger groups at baseline (IL-8, *p* = 0.819; MCP-1, *p* = 0.248; VEGF, *p* = 0.264). Epidermal growth factor (EGF) was greater in the older group compared to the younger group at baseline [142 (20) pg mL^−1^ and 60 (12) pg mL^−1^, respectively, *p* = 0.001, Cohen's *d* = 1.64]. Following SIT, older men decreased EGF to 100 (12) pg mL^−1^ which was similar to that of young men who did not undergo training (*p* = 0.113, Cohen's *d* = 1.07).

**Conclusion:**

Older aerobically trained men have greater serum EGF than younger aerobically trained men. A novel SIT intervention in older men can shift circulating EGF towards trained younger concentrations. As lower EGF has previously been associated with longevity in *C. elegans*, the manipulative effect of SIT on EGF in healthy ageing in the human may be of further interest.

## Introduction

Human ageing involves a loss of function of multiple physiological systems, including the cardiovascular system, respiratory system, musculoskeletal system, and immuno-senescence (Rebelo-Marques et al. 2018). Circulating cytokine dysregulation is well recognised as a consequence of biological ageing (Alvarez-Rodriguez et al. 2012). The 'inflamm-ageing' hypothesis suggests that chronic ageing is associated with increased reactive oxygen species and increased basal pro-inflammatory state (Franceschi et al. 2007). Indeed, tumour necrosis factor alpha (TNFα) is greater in 80-year-olds relative to younger individuals and greater again in centenarians. Similarly, interleukin (IL)-6 is elevated with increasing age (Bruunsgaard et al. 1999; Baylis et al. 2013; Kanikowska et al. 2014) while intracellular pro-inflammatory cytokines (including interferon gamma [IFNγ] and TNFα) are seen to be elevated in T cells of older vs young participants (Zanni et al. 2003).

The deleterious effects of ageing on immune function are linked to dysregulation of cytokines which are responsible for the promotion of the pro-ageing senescence-associated secretory phenotype (Coppé et al. 2010). It has been reported the senescence-associated secretory phenotype is promoted by excess body fat associated with increased pro-inflammatory adipokines and cytokines, such as IL-6 and IL-8, alongside cytokines such as monocyte chemoattractant protein-1 (MCP-1), IFNγ, and TNFα (Christiansen et al. 2005; Monzillo et al. 2003; Sharabiani et al. 2011; Vieira et al. 2009). This is further compounded by decreased anti-inflammatory myokine expression, which disrupts inflammatory balance, facilitating pathological developments, including insulin resistance, cardiovascular disease, sarcopenia, chronic kidney disease, neurodegenerative disease, and increased inflamm-ageing of all organs (Muller et al. 2019). Moreover, growth factors, such as vascular endothelial growth factor (VEGF) and epidermal growth factor (EGF), when overexpressed, facilitate increased autoimmune diseases activity and tumorigenesis (Shaik-Dasthagirisaheb et al. 2013; Kasza 2013). Concerning EGF specifically, Meybosch et al. (2019) noted significant inverse correlations between EGF (normalised for body surface area) and age, and EGF and body height. There was a notable and dramatic decrease in EGF post-puberty, causing authors to emphasise the importance of EGF in maturation and growth during the early years of life. What is unknown however, is the influence of physical fitness, physical activity levels, and exercise training on EGF.

Interestingly, whilst the ageing process is omnipresent in humans, physical activity can meaningfully attenuate the development of senescence-associated secretory phenotype (Garatachea et al. 2015). Masters athletes possess superior muscle and cardiovascular function relative to untrained age-matched individuals, but still show decreases in physiological function with increased age, suggesting lifelong exercise can delay, but not prevent, ageing-related changes to physiological systems, including inflammatory cytokine concentrations (Campbell et al. 2019; Duggal et al. 2018; Elliott et al. 2018; Ganse et al. 2018; Pollock et al. 2015).

Formalised physical activity, such as aerobic training and resistance training, have been widely researched for health promoting benefits in older populations (American College of Sports Medicine et al. 2009; Hayes et al. 2015; Hayes and Elliott 2019; Sellami et al. 2019, 2020). Previous reviews have found both aerobic and resistance training to be effective in attenuating senescence-associated secretory phenotype development (Muller et al. 2019; Sellami et al. 2018). Further, a review by Muller et al. (2019) suggests high-intensity interval training (HIIT) also attenuates the senescence-associated secretory phenotype. Previously described by MacInnis and Gibala (2017), HIIT utilises periods of high-intensity exercise interspersed by lower intensity phases of recovery. Generally, even with lower training volumes, HIIT produces similar health benefits when compared to classical forms of aerobic training, and has been deemed time-efficient and enjoyable in various populations (Gibala et al. 2012; Gillen and Gibala 2014; Hayes et al. 2020; Herbert et al. 2017; Hurst et al. 2019; Ramos et al. 2015; Weston et al. 2014). Although HIIT is effective in improving physiological function, it has been suggested the perceived difficulty of performing HIIT coupled with complex prescription may dissuade individuals from adopting HIIT (Biddle and Batterham 2015; Buchheit and Laursen 2013). Yet, a distinct derivative of HIIT, sprint interval training (SIT) offers an easier to prescribe exercise format (i.e. ‘all-out’). SIT has been described as enjoyable, tolerable, and easier to prescribe than HIIT, whilst still promoting positive physiological adaptations (MacInnis and Gibala 2017; Olney et al. 2018; Stork et al. 2018; Thum et al. 2017; Vollard et al. 2017; Vollard and Metcalfe 2017). Therefore, it is of interest to the field of exercise science and gerontology to investigate the effects of SIT on immune-modulating cytokines and growth factors (Hwang et al. 2020).

To separate the effect of ageing from any effect of lifelong inactivity on circulating pro-inflammatory cytokines, anti-inflammatory cytokines, and growth factors, we aimed to first establish the effect of age on circulating inflammatory markers and growth factors in well-trained young and older men, by comparing these biomarkers in a cohort of young men, and a cohort of older men who were all aerobically trained. A secondary aim was to examine the effect of a novel SIT stimuli on older aerobically trained men. It was hypothesised that older men would show elevated pro-inflammatory cytokines relative to a young cohort, and SIT would reduce pro-inflammatory cytokine concentrations.

## Methods

### Participants

**Fig. 1:**
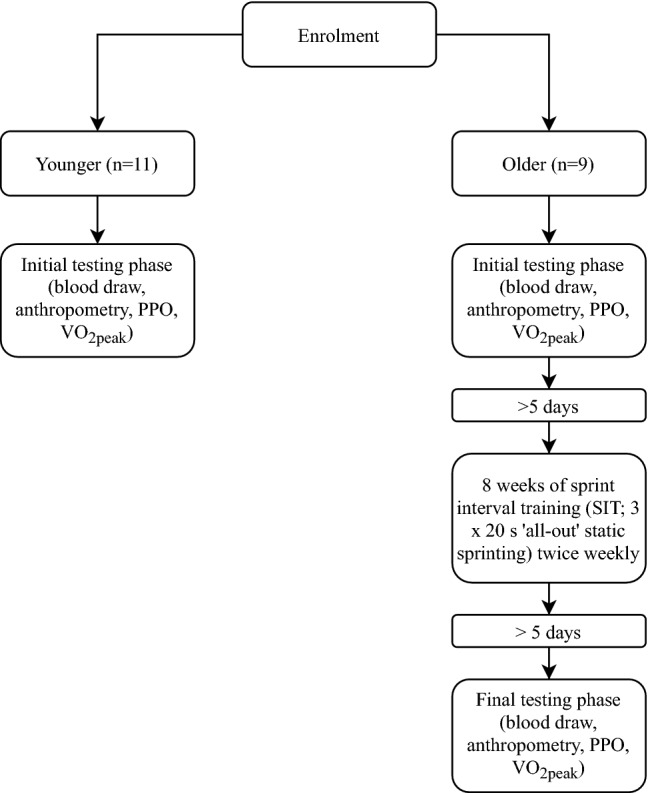
Schematic representation of the methodological flow. *PPO* peak power output, *VO*_*2peak*_ peak oxygen uptake

Two cohorts were recruited for this study, younger (*n* = 11; 21–34 years of age) and older (*n* = 9; 63–73 years of age) men, who regularly participated in a weekly minimum of 150 min.wk^−1^ of moderate- or high-intensity exercise for at least 6 months prior to participating in the study and continued habitual physical activity for the duration of the study. Participants were free of exercise contraindicating disease or injury as determined by a Physical Activity Readiness Questionnaire and American College of Sports Medicine pre-exercise participation screening (Riebe et al. 2015). This study was carried out in accordance with the Declaration of Helsinki and approved by the University of Cumbria Research Ethics Committee. Written informed consent was obtained from all participants prior to study commencement and subjects were excluded if they presented with atrial fibrillation. Descriptive statistics for participants are shown in Table 1: Participant anthropometric and performance parameters at baseline (young and older pre-training) and following sprint interval training (SIT; older post-training). Values given as mean (SD)., and further described in the Results section. Participants attended all sessions with exercise-suitable clothing and footwear. The younger cohort attended a single test session whilst the older cohort attended two separate testing sessions 5 days prior to, and 5 days after, the final SIT session of the intervention, which was 8 weeks in duration (Fig. 1).

**Table 1:** Participant anthropometric and performance parameters at baseline (young and older pre-training) and following sprint interval training (SIT; older post-training)

	Young (*n* = 11)	Older	
		Pre-SIT (*n* = 9)	Post-SIT (*n* = 9)
Age (years)	28 (5)	68 (3)^a^	–
BMI (kg m^−2^)	23 (2)	23 (3)	24 (3)^b^
VO_2peak_ (mL kg min^−1^)	55 (11)	39 (6)^a^	41 (8)
PPO (W)	1149 (131)	696 (89)^a^	727 (76)
Resting heart rate (b·min^−1^)	53 (10)	56 (7)	55 (7)
Systolic blood pressure (mmHg)	127 (10)	129 (16)	126 (14)
Diastolic blood pressure (mmHg)	77 (8)	77 (10)	77 (10)

Values given as mean (SD)

*SIT* sprint interval training, *BMI* body mass index, *VO*_*2peak*_ peak oxygen uptake, *PPO* peak power output

^a^Young different to older at the *p* < 0.05 level

^b^Older pre-SIT different to older post-SIT at the *p* < 0.05 level

### Blood draws and analysis

Participants arrived at the exercise physiology laboratory between 08.00 and 11.00 h, following an overnight fast and having abstained from strenuous physical activity for a minimum of 48 h. Participants were reminded to maintain standardised conditions prior to each assessment point which included arriving in a hydrated state having abstained from caffeine and alcohol consumption for 24 h. Following 20 min supine rest, blood was sampled from the antecubital vein using standard venepuncture method into sterile serum separator vacutainer tubes (Becton Dickinson, Rutherford, NJ) that were kept at room temperature in the dark, for 30 min, to allow for clotting, after which samples were centrifuged at 1100 g for 15 min. Serum was then extracted, aliquoted, and stored at − 80 °C until subsequent analysis. Blood samples were collected at the same time of day for each participant to control for biological variation and minimise inter-participant variation. Blood draws were completed prior to any exercise testing.

### Anthropometry

Height was measured to the nearest 0.1 cm, and mass to the nearest 0.01 kg using a Seca 286 measuring station (Birmingham, UK), from which body mass index (BMI) was derived by dividing mass by the square of height (kg/m^2^).

### Peak power output (PPO)

PPO was established using the 6 s Herbert test (Herbert et al. 2015) on an air-braked cycle ergometer (Wattbike Ltd., Nottingham, UK), which consisted of a maximal 6 s sprint from a standing start.

### Peak oxygen uptake (VO_2peak_)

At least five min after PPO determination, VO_2peak_ was determined using a Cortex II Metalyser 3B-R2 (Cortex, Biophysik, Leipzig, Germany). Expiratory airflow was achieved using a volume transducer (Triple V® turbine, digital) connected to an oxygen (O_2_) analyser. Expired gases were analysed for O_2_ with electrochemical cells and for carbon dioxide CO_2_ output with an infrared analyser. The Metalyser was calibrated according to manufacturer's guidelines prior to each test. After a 60 min warm-up period, the O_2_ and CO_2_ sensors were calibrated against environmental air in addition to reference gas of known composition (5% CO_2_, 15% O_2_, and 80% N_2_) with volume calibrated by five inspiratory and expiratory strokes using a 3 L pump. Prior to determination of VO_2peak_, a chest strap heart rate monitor was attached to participants' chests, with heart rate measured continuously throughout the test (Polar F1, Polar, Finland). The cycle ergometer (Wattbike Pro, Wattbike, UK) was adjusted to manufacturer’s guidance. Saddle height was adjusted relative to the crank position and the foot was secured to a pedal with straps with participants' knee at almost full extension (~ 170°). Participants mounted the cycle ergometer, and a rubber face mask was fitted (Hans Rudolph Inc, USA), which was attached to the Cortex II Metalyser 3B-R2. VO_2_ and VCO_2_ were recorded continuously throughout the test.

Participants completed a 3 min warm-up at an intensity equivalent to ~ 10% of PPO. Subsequently, participants cycled at increasing intensity with 25 W increments each min until they reached volitional exhaustion, with rating of perceived exertion [RPE; 0–10 scale; Borg (1998)] recorded in the last 10 s of each stage. Immediately following volitional exhaustion, participants had their index finger cleaned using a disinfectant wipe, and then a lancet was used to lacerate the fingertip to obtain a blood sample for to measure blood lactate (Lactate Pro 2, Arkray, Japan). VO_2peak_ was confirmed when participants achieved a minimum of any four of the following criteria; VO_2_ plateau, RER ≥ 1.10, peak heart rate within 10 beats of age predicted maximum and [BLa] ≥ 8 mmol·L^−1^, final RPE of ≥ 9.

### Cytokine array

Cytokine concentrations were quantified in an aliquot of serum utilizing a chip array system (Cytokine array I, Evidence Investigator, Affinity Biolabs, UK) with a sandwich chemiluminescent immunoassay technique for epidermal growth factor (EGF), interleukins (IL-1a, -1b, -2, -4, -6, -8, -10), IFN-γ, MCP-1, TNFα, and VEGF. Method precision and lower/upper limits of sensitivity have been previously reported (Karuppasamy et al. 2011), and quality controls were performed by the manufacturer using three known concentrations for each cytokine.

### Exercise training

**Fig. 2:**
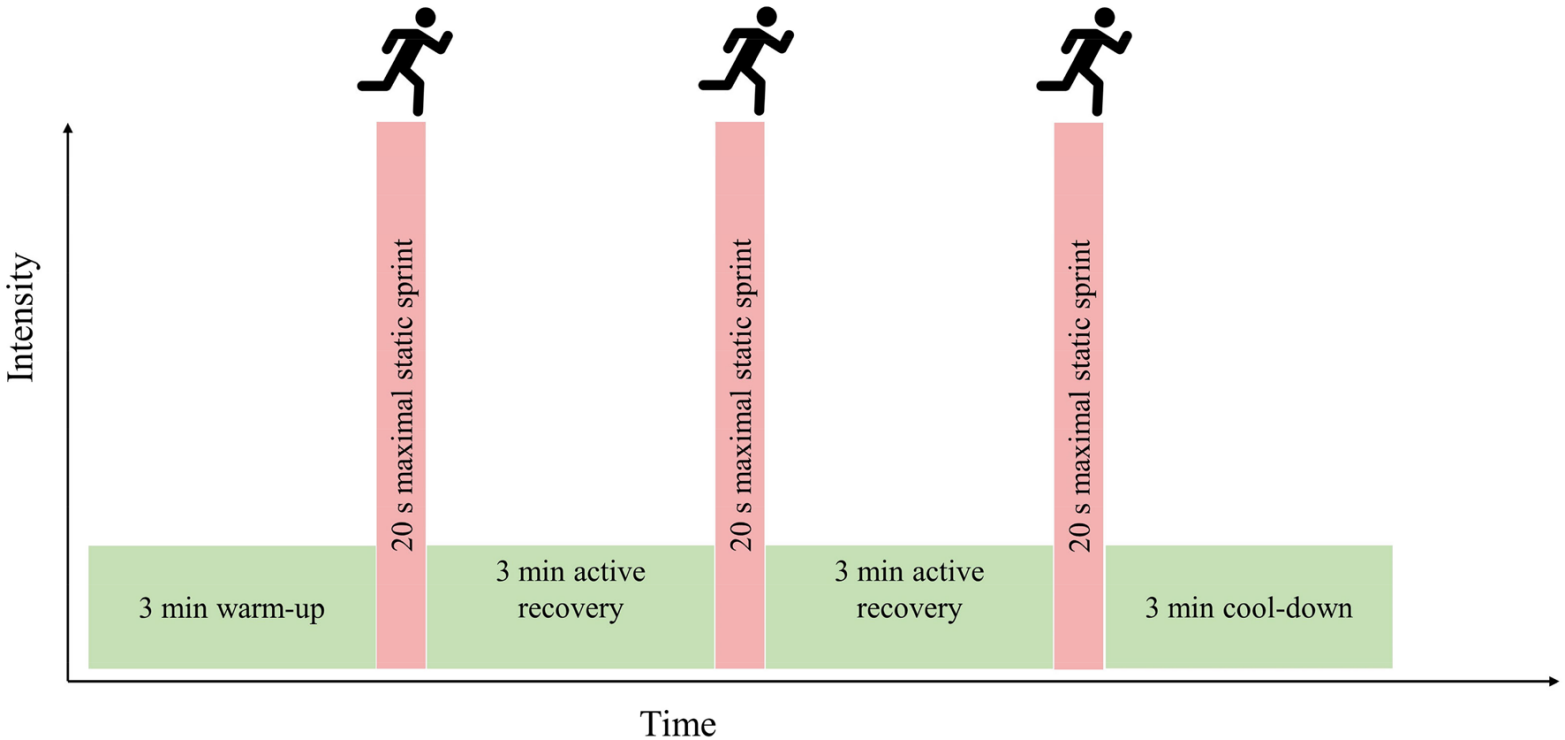
Schematic representation of the sprint interval session. Participants performed this session twice weekly for eight weeks

Older participants attended two SIT sessions per week, 72 h apart, as our pilot work suggested older adults would be suitably recovered from SIT in this timeframe (Yasar et al. 2019). Participants avoided strenuous physical activity 24 h prior to SIT sessions whilst maintaining habitual physical activity according to self-reporting. Participants warmed up for a period of 3 min at a self-paced intensity by performing static running. Participants then performed three 20 s static sprints at an ‘all-out’ intensity, interspersed by 3 min self-paced recovery phases. Following the final sprint, a 3 min self-paced cool down was performed (Fig. 2). During all sprints, participants were instructed to raise their feet to approximately knee height, with loud verbal encouragement throughout each sprint.

### Statistical analysis

Following confirmation of normality by a D’Agostino & Pearson normality test, cytokine data were examined by one-way analysis of variance (ANOVA) or Kruskal–Wallis test as appropriate, with post hoc interrogation by Dunnett’s multiple comparison test (younger as comparison group). Descriptive statistics (younger vs older pre-training) and training effects (older group only) were examined by unpaired t test or Mann–Whitney test as appropriate. Fisher's exact test tested for dichotomous differences in whether a cytokine was above or below the minimum level of detection in the older and younger group. Relationships between variables were determined using Pearson’s product-moment correlation coefficient. Effect size for paired comparisons is reported as Cohen’s *d*, interpreted as trivial (< 0.20), small (≥ 0.20–0.49), moderate (≥ 0.50–0.79), and large (≥ 0.80). Parametric data sets are summarised in text as mean and standard deviation (SD) whilst non-parametric are given as median (upper–lower quartile). Figures are presented as grouped dot plots, as recommended by Drummond and Vowler (2011). Alpha level was not set dichotomously as significant or non-significant as recommended by Hurlbert et al. (2019). All figures were generated in GraphPad (5.02, GraphPad Software, USA) or R [version 3.6.1, (R Core Team (2019))] utilizing the *Hmisc* (Harrell et al. 2020) and the *corrplot* (Wei and Simko 2017) packages.

## Results

### Anthropometric and performance measures

At baseline, older men did not differ from younger men in terms of body mass (*p* = 0.635, Cohen's *d* = 0.13), BMI (*p* = 0.070, Cohen's *d* = 0.04) resting heart rate BMI (*p* = 0.517, Cohen's *d* = 0.30), systolic blood pressure BMI (*p* = 0.803, Cohen's *d* = 0.11), diastolic blood pressure BMI (*p* = 0.896, Cohen's *d* = 0.06), or BMI (*p* = 0.070, Cohen's *d* = 0.04). However, older men did exhibit a lower VO_2peak_ (*p* = 0.004, Cohen's *d* = 1.48) and PPO (*p* < 0.001 Cohen’s *d* = 4.05; Table 1). The SIT intervention produced a trivial increase in older participants' BMI (*p* = 0.039, Cohen's *d* = 0.12), a small increase in VO_2peak_ (*p* = 0.268, Cohen's *d* = 0.23), a small increase in PPO (*p* = 0.072, Cohen's *d* = 0.35), a small decrease in resting heart rate (*p* = 0.263, Cohen’s *d* = 0.40) a trivial reduction in systolic blood pressure (*p* = 0.701, Cohen's *d* = 0.13), and a small decrease in diastolic blood pressure (*p* = 0.347, Cohen’s *d* = 0.33).

### Cytokines

Of the 12 cytokines measured by chip array, IL-1a, IL-1b, IL-2, IL-4, IL-6, IL-10, IFN-γ and TNFα were frequently below the limit of detection of array methodology, and thus concentrations are not further reported. For clarity, we report on cytokines whereby > 75% of samples returned with values above the lower limit of detection. Ordinal analysis of the data suggests that pro-inflammatory cytokines IL-1a, IL-1b, IL-6 were more frequently observed in the older cohort, whilst classically anti-inflammatory cytokines IL-2 and IL-10 were more often observed quantifiable in the younger cohort. However, Fisher's exact test revealed no differences between younger and older for the frequency of cytokines above or below the limit of detection (Table 2). Pro-inflammatory cytokines IL-8 and MCP-1, and growth factors VEGF and EGF were consistently detected and further described below.

**Table 2:** Cytokine marker state at baseline for young (*n* = 11) and older (*n* = 9)

Cytokine	Young *N* = 11	Older *N* = 9	Lower limit of detection (pg mL^−1^)	Accepted (y/n)	*p* value
EGF	11	9	2.9	Yes	1.000
IL-1a	4	5	0.8	No	0.653
IL-1b	3	4	1.6	No	0.642
IL-2	3	0	4.8	No	0.218
IL-4	0	0	6.6	No	1.000
IL-6	4	6	1.2	No	0.370
IL-8	10	7	4.9	Yes	0.569
IL-10	2	0	1.8	No	0.479
IFN-γ	0	0	3.5	No	1.000
MCP-1	11	9	13.2	Yes	1.000
TNFα	0	0	4.4	No	1.000
VEGF	10	9	14.6	Yes	1.000

Markers were accepted if > 75% of samples returned concentrations > lower limit of detection. *p* values represent Fisher's exact test for whether the proportion of cytokine detected was different between the young and older group

**Fig. 3:**
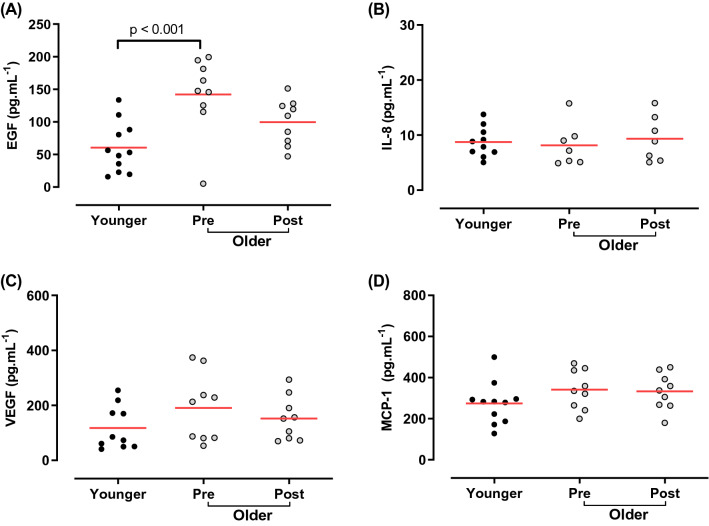
Cytokine concentrations of young, older pre- and older post-sprint interval training. **a** EGF, **b** IL-8, **c** VEGF and **d** MCP-1. Young shown in black circles, older shown in grey. Red horizontal lines indicate group means

The effect of age and SIT on EGF, IL-8, VEGF and MCP-1, was compared by one-way [condition (younger, older pre-training, older post-training)] ANOVA. EGF showed an effect of condition (*p* = 0.002). The effect of condition was examined post hoc by Dunnett’s multiple comparison test, with the younger condition as the comparison. Older pre-training EGF was higher compared to the younger group (*p* = 0.001, Cohen's *d* = 1.64; Fig. 3), whilst the older post-training values were the same as the younger group [*p* = 0.113, Cohen's *d* = 1.07; younger 60 (12) pg mL^−1^, older pre-training 142 (20) pg mL^−1^, older post-training 100 (12) pg mL^−1^]. There was a large decrease in EGF in the older cohort as a result of SIT (*p* = 0.101, Cohen's *d* = 0.87). There was no effect of group on remaining pro-inflammatory cytokines [IL-8, *p* = 0.819, Cohen's *d* = 0.28; younger 9 (3) pg mL^−1^, older pre-training 8 (4) pg mL^−1^, older post-training 9 (4) pg mL^−1^; MCP-1, *p* = 0.248, Cohen's *d* = 0.68; younger 274 (102) pg mL^−1^, older pre-training 341 (95) pg mL^−1^, older post-training 333 (88) pg mL^−1^] or VEGF [*p* = 0.264, Cohen's *d* = 0.72; younger 117 (79) pg mL^−1^, older pre-training 191 (123) pg mL^−1^, older post-training 152 (80) pg mL^−1^; Fig. 3b–d]. When examining the magnitude of effect of training in the older group, there was a trivial effect of SIT on MCP-1 (*n* = 9; Cohen's *d* = 0.09), and a small increase in IL-8 (*n* = 7; Cohen's *d* = 0.30) and a small decrease in VEGF (*n* = 9; Cohen's *d* = 0.38).

**Fig. 4:**
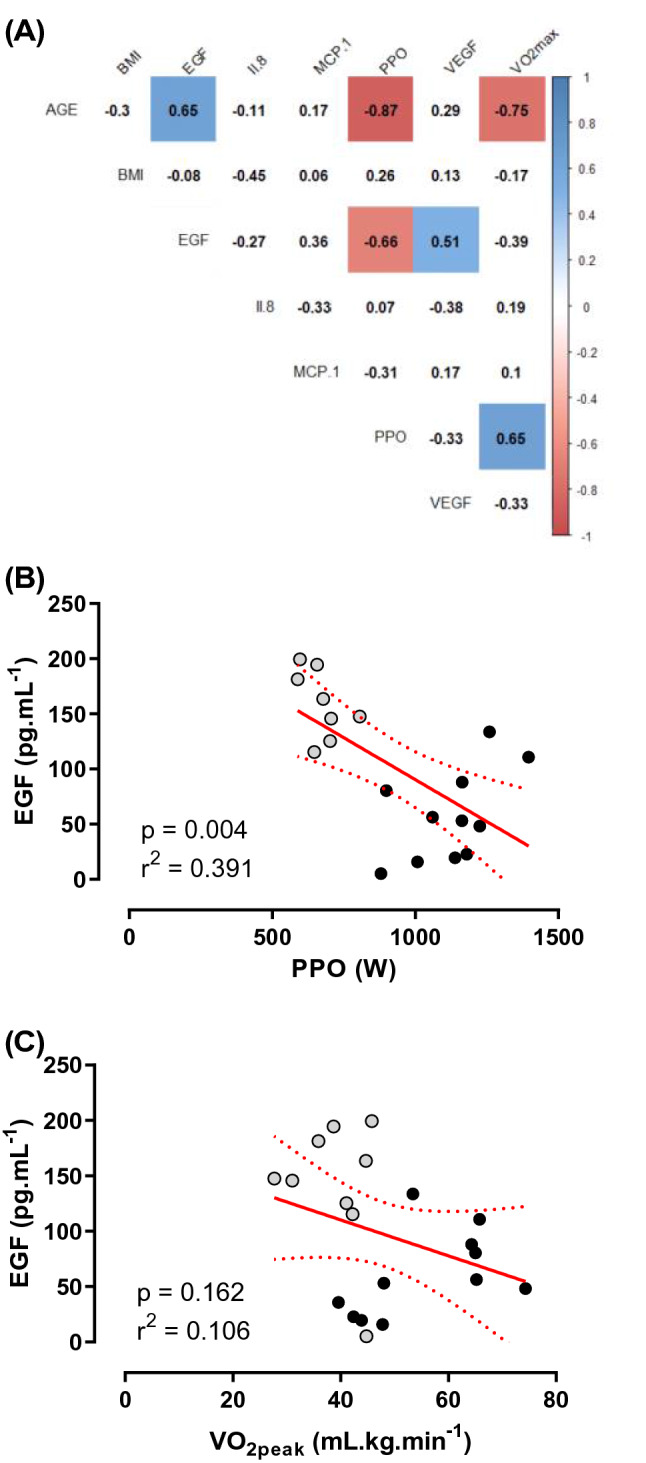
Correlations between physiological and cytokine markers. **a** Correlation matrix where values indicate r correlation coefficient and filled squares indicate where *p* < 0.05. Shading indicates strength of relationship (blue = positive, red = negative correlation). **b** EGF (pg mL^−1^) as a function of PPO (W), **c** EGF (pg mL^−1^) as a function of VO_2peak_ (mL kg min^−1^). For both (**b**) and (**c**), linear correlation indicated by red line, 95% confidence indicated by red dashed lines. Grey circles indicate older, black indicates younger

Relationships between baseline characteristics and circulating cytokines were examined by Pearson’s correlation matrix (Fig. 4a). Age was strongly and negatively correlated with PPO and VO_2peak_, and moderately associated with EGF (Fig. 4b). The EGF-PPO relationship was moderate (*p* = 0.004, *r*^2^ = 0.391; Fig. 3b), and the EGF-VO_2peak_ relationship was weak (*p* = 0.162, *r*^2^ = 0.106; Fig. 4c).

## Discussion

The primary findings from the present study were (1) baseline EGF was greater in trained older men compared to younger participants, (2) there was no baseline differences in most (IL-1a, IL-1b, IL-2, IL-6, IL-8, IFN-γ, MCP-1, and TNFα) pro-inflammatory cytokines between trained older men and trained younger men, and (3) we make the novel observation that EGF was reduced to levels of younger men by a novel 8-week SIT intervention in trained older men.

Of the cytokines measured in the present work, only EGF was different between younger and older at baseline. EGF has a well-understood action via the activation of the EGF receptor which is linked to inflammatory responses in terms of wound healing in mouse model keratinocytes, cellular proliferation, chronic kidney disease and tumorigenesis in humans, all of which are negative outcomes of ageing (Choi et al. 2018; Kasza 2013; Rayego-Mateos et al. 2018). However, data presented here should not be read as support of EGF as an activity-independent marker of biological age, as the addition of a novel exercise stimulus reduced EGF concentration in older participants. Indeed, it has been previously shown that overweight sedentary individuals possess lower plasma EGF compared to normal weight controls (Accattato et al. 2017). What physiological effect these alterations in EGF have on healthspan and lifespan can only be speculated at with the data presented here, but it is interesting to observe that a gain-of-function mutation in the EGF receptor promotes longevity in the model organism *C. elegans*, whilst loss-of-function mutations negatively affect longevity (Iwasa et al. 2010; Rongo 2011; Siddiqui et al. 2012).

We demonstrated 8 weeks of SIT-reduced EGF in SIT-naïve, but aerobically trained older men. We are unaware of other studies that investigate the effect of exercise training (i.e. > 1 month) on EGF in older men. However, Accattato et al. (2017) established a single bout of endurance exercise (20 min run at 70% VO_2peak_) acutely suppresses EGF in younger individuals, yet resistance training has been shown to acutely increase EGF in healthy trained men (Diaz-Castro et al. 2020). Thus, it is clear the type of exercise (resistance vs endurance) influences EGF response after a period of training as recent studies in C2C12 myotubes have shown that EGF receptor inhibition promotes a slow twitch (oxidative) over a fast-twitch muscle phenotype (Ciano et al. 2019). Thus, after resistance training, an increase in EGF would be associated with an increase in muscle protein synthesis and hypertrophy whereas a decrease in EGF after endurance exercise is associated with oxidative adaptation. The clinical significance of these changes in EGF following exercise training is unclear however. Whilst greater EGF receptor prevalence is associated with multiple cancer types (Fisher et al. 2018; Gao et al. 2016; Tokunaga et al. 1995), cardiovascular disease (Makki et al. 2013), and in vitro EGF has been shown to influence cellular proliferation and differentiation rates [included in C2C12 myocytes (Ciano et al. 2019)], it is difficult to speculate concerning the biological role that post-SIT EGF suppression exerts in older men here.

Ageing is associated with a fast-to-slow muscle fibre type shift (Brunner et al. 2007; Deschenes 2004), as is chronic endurance training (Hawley et al. 2014), and this observation is maintained in lifelong endurance-trained older individuals (Dubé et al. 2016). In a cohort of both healthy controls and chronic obstructive pulmonary disease patients, greater muscle EGF messenger ribonucleic acid (mRNA) expression was associated with fewer slow-twitch muscle fibres and lower VO_2peak_ (Ciano et al. 2019). Interestingly, our data suggest lifelong endurance training into older age is associated with higher EGF expression than younger adults, yet a relatively high VO_2peak_. The reasonably expected large percentage of slow-twitch fibre type expression in our trained older participants may correlate with higher EGF expression, and the introduction of a ‘fast twitch’ promoting training stimulus could thus be speculated to induce the witnessed depression in circulating EGF, yet muscle biopsies would be required to confirm the fibre type shift.

Ageing is associated with an increased basal expression of circulating pro-inflammatory cytokines (Michaud et al. 2013). A recent meta-analysis concluded that chronic (at least 4 weeks) aerobic exercise in middle aged and older individuals decreased pro-inflammatory markers TNFα and IL-6 (Zheng et al. 2019). In addition, low physical activity levels and high sitting time increase overall risk of death from inflammation-related chronic disorders in people aged > 60 years (Cabanas-Sanchez et al. 2018). In line with this, our results demonstrate that aerobically trained older men possess low circulating concentrations of several pro-inflammatory cytokines. Our data are thus in line with the hypothesis that basal inflammation seen in older individuals may be partly inactivity-induced, and not a result of chronological ageing per se. This is supported by the fact that several of the cytokines reported here were below assay limits of detection, our participants did not show the elevated systemic inflammation typically seen in inactive older populations.

VEGF is a potent angiogenetic factor (Apte et al. 2019) and is essential for exercise-induced angiogenesis and subsequent improvements in performance (Wagner et al. 2006). In younger adults, resting VEGF was not changed following a HIIT intervention of 6 weeks (Żebrowska et al. 2019). VEGF positively associates with age in adults (Ruggiero et al. 2011) and has previously been reported to be increased in sedentary older individuals relative to lifelong exercisers, and further increased in sedentary individuals by 6 weeks of HIIT (Grace et al. 2015). We see no difference either in younger vs older trained individuals, or any pre-to-post training effect in our older population. Thus, any effects of ageing on circulated VEGF may be negated by lifelong exercise behaviour. In a similar manner, MCP-1 positivity associates with age in mice and is elevated in older frail individuals relative to non-frail age-matched controls (Yousefzadeh et al. 2018). As MCP-1 was not elevated in our cohort of trained older individuals relative to our younger population, this provides further support of the use of MCP-1 and VEGF as a marker of biological age, however, the addition of an inactive ageing control group to our model is needed to confirm this.

Some limitations to our study design should be acknowledged. We specifically sought to examine trained older individuals, comparing them to trained younger adults to remove any effect of inactivity on ageing. However, the addition of an inactive older group would have been a useful addition to confirm inactivity-associated ageing changes in pro-inflammatory cytokines and growth factors that others have reported. Likewise, a young training group would have provided insight as to whether they possess more plasticity with regards to serum cytokine concentrations. In addition, this study did not include women, and therefore, findings cannot be extrapolated to women. Having multiple cytokine markers below useful limits of detection was a methodological weakness of the approach that we have utilised here, and future studies will need to consider the use of high-sensitivity biochip cytokine arrays, individual ELISA per marker, or the use of multiplex ELISA techniques, however, these methodological approaches are associated with greater resource commitments. In addition, the present study did not verify objectively measured physical activity of participants during the study. Instead, the present study relied on self-reporting, which is subject to self-reporting bias.

In conclusion, here we make novel observations on the state of circulating pro- and anti-inflammatory markers in trained older individuals. EGF was greater in endurance trained older individuals compared to younger men, however, the addition of a novel SIT intervention in older men can shift circulating EGF towards trained younger concentrations. As EGF has previously been associated with longevity in *C. elegans*, the manipulative effect of SIT on EGF in healthy ageing in the human may be of further interest.

## Abbreviations

ANOVA: Analysis of variance
BLa: Blood lactate
BMI: Body mass index
EGF: Epidermal growth factor
HIIT: High-intensity interval training
IFNγ: Interferon gamma
IL: Interleukin
MCP-1: Monocyte chemoattractant protein-1
mRNA: Messenger ribonucleic acid
N_2_: Nitrogen
O_2_: Oxygen
PPO: Peak power output
RER: Respiratory exchange ratio
RPE: Rating of perceived exertion
SD: Standard deviation
SIT: Sprint interval training
TNFα: Tumour necrosis factor alpha
VEGF: Vascular endothelial growth factor
VO_2_: Oxygen uptake
VO_2peak_: Peak oxygen uptake

## References

[CR1] Accattato F, Greco M, Pullano SA, Carè I, Fiorillo AS, Pujia A, Montalcini T, Foti DP, Brunetti A, Gulletta E (2017). Effects of acute physical exercise on oxidative stress and inflammatory status in young, sedentary obese subjects. PLoS ONE.

[CR2] Álvarez-Rodríguez L, López-Hoyos M, Muñoz-Cacho P, Martínez-Taboada VM (2012). Aging is associated with circulating cytokine dysregulation. Cell Immunol.

[CR95] Chodzko-Zajko WJ, Proctor DN, Fiatarone Singh MA, Minson CT, Nigg CR, Salem GJ, Skinner JS, American College of Sports Medicine (2009). American College of Sports Medicine position stand. Exercise and physical activity for older adults. Med Sci Sports Exerc.

[CR3] Apte RS, Chen DS, Ferrara N (2019). VEGF in signaling and disease: Beyond discovery and development. Cell.

[CR4] Baylis D, Bartlett DB, Patel HP, Roberts HC (2013). Understanding how we age: insights into inflammaging. Longev Healthspan.

[CR90] Biddle SJ, Batterham AM (2015). High-intensity interval exercise training for public health: a big HIT or shall we HIT it on the head?. Int J Behav Nutr Phys Act.

[CR5] Borg G (1998). Borg’s perceived exertion and pain scales.

[CR6] Brunner F, Schmid A, Sheikhzadeh A, Nordin M, Yoon J, Frankel V (2007). Effects of aging on Type II muscle fibers: a systematic review of the literature. J Aging Phys Act.

[CR7] Bruunsgaard H, Andersen-Ranberg K, Jeune B, Pedersen AN, Skinhøj P, Pedersen BK (1999). A high plasma concentration of TNF-alpha is associated with dementia in centenarians. J Gerontol A Biol Sci Med Sci.

[CR8] Buchheit M, Laursen PB (2013). High-intensity interval training, solutions to the programming puzzle: part I: cardiopulmonary emphasis. Sports Med.

[CR9] Cabanas-Sánchez V, Guallar-Castillón P, Higueras-Fresnillo S, García-Esquinas E, Rodríguez-Artalejo F, Martinez-Gomez D (2018). Physical activity, sitting time, and mortality from inflammatory diseases in older adults. Front Physiol.

[CR10] Campbell A, Grace F, Ritchie L, Beaumont A, Sculthorpe N (2019). Long-term aerobic exercise improves vascular function into old age: a systematic review, meta-analysis and meta regression of observational and interventional studies. Front Physiol.

[CR11] Choi SY, Lee YJ, Kim JM, Kang HJ, Cho SH, Chang SE (2018). Epidermal growth factor relieves inflammatory signals in staphylococcus aureus-treated human epidermal keratinocytes and atopic dermatitis-like skin lesions in Nc/Nga mice. Biomed Red Int.

[CR12] Christiansen T, Richelsen B, Bruun JM (2005). Monocyte chemoattractant protein-1 is produced in isolated adipocytes, associated with adiposity and reduced after weight loss in morbid obese subjects. Int J Obes.

[CR13] Ciano M, Mantellato G, Connolly M, Paul-Clark M, Willis-Owen S, Moffatt MF, Cookson WOCM, Mitchell JA, Polkey MI, Hughes SM, Kemp PR, Natanek SA (2019). EGF receptor (EGFR) inhibition promotes a slow-twitch oxidative, over a fast-twitch, muscle phenotype. Sci Rep.

[CR14] Coppé J-P, Desprez P-Y, Krtolica A, Campisi J (2010). The senescence-associated secretory phenotype: the dark side of tumor suppression. Annu Rev Pathol.

[CR15] Deschenes MR (2004). Effects of aging on muscle fibre type and size. Sports Med.

[CR16] Diaz-Castro J, Moreno-Fernandez J, Chirosa I, Chirosa LJ, Guisado R, Ochoa JJ (2020). Beneficial effect of ubiquinol on hematological and inflammatory signaling during exercise. Nutrients.

[CR17] Drummond GB, Vowler SL (2011). Show the data, don't conceal them. Br J Pharmacol.

[CR18] Dubé JJ, Broskey NT, Despines AA, Stefanovic-Racic M, Toledo FGS, Goodpaster BH, Amati F (2016). Muscle characteristics and substrate energetics in lifelong endurance athletes. Med Sci Sports Exerc.

[CR19] Duggal NA, Pollock RD, Lazarus NR, Harridge S, Lord JM (2018). Major features of immunesenescence, including reduced thymic output, are ameliorated by high levels of physical activity in adulthood. Aging Cell.

[CR20] Elliott BT, Herbert P, Sculthorpe N, Grace FM, Stratton D, Hayes LD (2018). Lifelong exercise, but not short-term high-intensity interval training, increases GDF11, a marker of successful aging: a preliminary investigation. Physiol Rep.

[CR21] Fisher SA, Tam YT, Fokina A, Mahmoodi MM, Distefano MD, Schoichet MS (2018). Photo-immobilized EGF chemical gradients differentially impact breast cancer cell invasion and drug response in defined 3D hydrogels. Biomaterials.

[CR22] Franceschi C, Capri M, Monti D, Giunta S, Olivieri F, Sevini F, Panourgia MP, Invidia L, Celani L, Scurti M, Cevenini E, Castellani GC, Salvioli S (2007). Inflammaging and anti-inflammaging: a systemic perspective on aging and longevity emerged from studies in humans. Mech Ageing Dev.

[CR23] Ganse B, Ganse U, Dahl J, Degens H (2018). Linear decrease in athletic performance during the human life span. Front Physiol.

[CR24] Gao L, Wang FQ, Li HM, Yang JG, Ren JG, He KF, Liu B, Zhang W, Zhao YF (2016). CCL2/EGF positive feedback loop between cancer cells and macrophages promotes cell migration and invasion in head and neck squamous cell carcinoma. Oncotarget.

[CR25] Garatachea N, Pareja-Galeano H, Sanchis-Gomar F, Santos-Lozano A, Fiuza-Luces C, Morán M, Emanuele E, Joyner MJ, Lucia A (2015). Exercise attenuates the major hallmarks of aging. Rejuvenation Res.

[CR26] Gibala MJ, Little JP, Macdonald MJ, Hawley JA (2012). Physiological adaptations to low-volume, high-intensity interval training in health and disease. J Physiol.

[CR27] Gillen JB, Gibala MJ (2014). Is high-intensity interval training a time-efficient exercise strategy to improve health and fitness?. Appl Physiol Nutr Metab.

[CR28] Grace FM, Herbert P, Ratcliffe JW, New KJ, Baker JS, Sculthorpe NF (2015). Age related vascular endothelial function following lifelong sedentariness: positive impact of cardiovascular conditioning without further improvement following low frequency high intensity interval training. Physiol Rep.

[CR29] Harrell FE with contributions from Charles Dupont and many others (2020) Hmisc: Harrell Miscellaneous. R package version 4.4–0. https://CRAN.R-project.org/package=Hmisc&&

[CR30] Hawley JA, Hargreaves M, Joyner MJ, Zierath JR (2014). Integrative biology of exercise. Cell.

[CR31] Hayes LD, Elliott BT (2019). Short-term exercise training inconsistently influences basal testosterone in older men: a systematic review and meta-analysis. Front Physiol.

[CR32] Hayes LD, Sculthorpe N, Herbert P, Baker JS, Spagna R, Grace FM (2015). Six weeks of conditioning exercise increases total, but not free testosterone in lifelong sedentary aging men. Aging Male.

[CR33] Hayes LD, Herbert P, Sculthorpe N, Grace F (2020). High intensity interval training (HIIT) produces small improvements in fasting glucose, insulin, and insulin resistance in sedentary older men but not masters athletes. Exp Gerontol.

[CR34] Herbert P, Sculthorpe N, Baker JS, Grace FM (2015). Validation of a six second cycle test for the determination of peak power output. Res Sports Med.

[CR35] Herbert P, Hayes LD, Sculthorpe NF, Grace FM (2017). HIIT produces increases in muscle power and free testosterone in male masters athletes. Endocr Conn.

[CR36] Hurlbert SH, Levine RA, Utts J (2019). Coup de grâce for a tough old bull: “statistically significant” expires. Am Stat.

[CR37] Hurst C, Weston KL, Weston M (2019). The effect of 12 weeks of combined upper- and lower-body high-intensity interval training on muscular and cardiorespiratory fitness in older adults. Aging Clin Exp Res.

[CR38] Hwang JH, McGovern J, Minett GM, Della Gatta PA, Roberts L, Harris JM, Thompson EW, Parker TJ, Peake JM, Neubauer O (2020). Mobilizing serum factors and immune cells through exercise to counteract age-related changes in cancer risk. Exerc Immunol Rev.

[CR39] Iwasa H, Yu S, Xue J, Driscoll M (2010). Novel EGF pathway regulators modulate *C. elegans* healthspan and lifespan via EGF receptor, PLC-gamma, and IP3R activation. Aging Cell.

[CR40] Kanikowska D, Pyda M, Korybalska K, Grajek S, Lesiak M, Bręborowicz A, Witowski J (2014). Age-related limitations of interleukin-6 in predicting early mortality in acute ST-elevation myocardial infarction. Immun Ageing.

[CR41] Karuppasamy P, Chaubey S, Dew T, Musto R, Sherwood R, Desai J, John L, Shah AM, Marber MS, Kunst G (2011). Remote intermittent ischemia before coronary artery bypass graft surgery: a strategy to reduce injury and inflammation?. Basic Res Cardiol.

[CR42] Kasza A (2013). IL-1 and EGF regulate expression of genes important in inflammation and cancer. Cytokine.

[CR43] MacInnis MJ, Gibala MJ (2017). Physiological adaptations to interval training and the role of exercise intensity. J Physiol.

[CR44] Makki N, Thiel KW, Miller FJ (2013). The epidermal growth factor receptor and its ligands in cardiovascular disease. Int J Mol Sci.

[CR45] Meybosch S, De Monie A, Anne C, Bruyndonckx L, Jurgens A, De Winter BY, Trouet D, Ledeganck KJ (2019). Epidermal growth factor and its influencing variables in healthy children and adults. PLoS ONE.

[CR46] Michaud M, Balardy L, Moulis G, Gaudin C, Peyrot C, Vellas B, Cesari M, Nourhashemi F (2013). Proinflammatory cytokines, aging, and age-related diseases. J Am Med Dir Assoc.

[CR47] Monzillo LU, Hamdy O, Horton ES, Ledbury S, Mullooly C, Jarema C, Porter S, Ovalle K, Moussa A, Mantzoros CS (2003). Effect of lifestyle modification on adipokine levels in obese subjects with insulin resistance. Obes Res.

[CR48] Muller L, Di Benedetto S, Pawelec G (2019). The immune system and its dysregulation with aging. Subcell Biochem.

[CR94] Olney N, Wertz T, LaPorta Z, Mora A, Serbas J, Astorino TA (2018). Comparison of acute physiological and psychological responses between moderate-intensity continuous exercise and three regimes of high-intensity interval training. J Strength Cond Res.

[CR49] Pollock RD, Carter S, Velloso CP, Duggal NA, Lord JM, Lazarus NR, Harridge SDR (2015). An investigation into the relationship between age and physiological function in highly active older adults. J Physiol.

[CR50] R Core Team (2019) R: A language and environment for statistical computing. R Foundation for Statistical Computing, Vienna, Austria. https://www.R-project.org/

[CR51] Ramos JS, Dalleck LC, Tjonna AE, Beetham KS, Coombes JS (2015). The impact of high-intensity interval training versus moderate-intensity continuous training on vascular function: a systematic review and meta-analysis. Sports Med.

[CR52] Rayego-Mateos S, Rodrigues-Diez R, Morgado-Pascual JL, Valentijn F, Valdivielso JM, Goldschmeding R, Ruiz-Ortega M (2018). Role of epidermal growth factor receptor (EGFR) and its ligands in kidney inflammation and damage. Mediat Inflamm.

[CR53] Rebelo-Marques A, De Sousa LA, Andrade R, Ribeiro CF, Mota-Pinto A, Carrilho F, Espregueira-Mendes J (2018). Aging hallmarks: the benefits of physical exercise. Front Endocrinol.

[CR54] Riebe D, Franklin BA, Thompson PD, Garber CE, Whitfield GP, Magal M, Pescatello LS (2015). Updating ACSM’s recommendations for exercise preparticipation health screening. Med Sci Sports Exerc.

[CR55] Rongo C (2011). Epidermal growth factor and aging: a signaling molecule reveals a new eye opening function. Aging.

[CR56] Ruggiero D, Dalmasso C, Nutile T, Sorice R, Dionisi L, Aversano M, Bröet P, Leutenegger A-L, Bourgain C, Ciullo M (2011). Genetics of VEGF serum variation in human isolated populations of cilento: Importance of VEGF polymorphisms. PLoS ONE.

[CR57] Sellami M, Guasmi M, Denham J, Hayes LD, Stratton D, Padulo J, Bragazzi NL (2018). Effects of acute and chronic exercise on immunological parameters in the elderly aged: Can physical activity counteract the effects of aging?. Front Immunol.

[CR58] Sellami M, Bragazzi NL, Slimani MHLD, Jabbour G, De Giorgio A, Dugue B (2019). The effect of exercise on glucoregulatory hormones: a countermeasure to human aging: Insights from a comprehensive review of the literature. Int J Environ Res Public Health.

[CR59] Sellami M, Abderrahmen AB, Dhabi W, Hayes LD, Zouhal H (2020). Hemoglobin, hematocrit and plasma volume variations following combined sprint and strength: effect of advanced age. Sci Sports.

[CR96] Shaik-Dasthagirisaheb YB, Varvara G, Murmura G, Saggini A, Caraffa A, Antinolfi P, Tete' S, Tripodi D, Conti F, Cianchetti E, Toniato E, Rosati M, Speranza L, Pantalone A, Saggini R, Tei M, Speziali A, Conti P, Theoharides TC, Pandolfi F (2013). Role of vitamins D, E and C in immunity and inflammation. J Biol Regul Homeost Agents.

[CR100] Sharabiani MT, Vermeulen R, Scoccianti C, Hosnijeh FS, Minelli L, Sacerdote C, Palli D, Krogh V, Tumino R, Chiodini P, Panico S, Vineis P (2011). Immunologic profile of excessive body weight. Biomarkers.

[CR60] Siddiqui S, Fang M, Ni B, Lu D, Martin B, Maudsley S (2012). Central role of the EGF receptor in neurometabolic aging. Int J Endocrinol.

[CR61] Stork MJ, Gibala MJ, Martin Ginis KA (2018). Psychological and behavioral responses to interval and continuous exercise. Med Sci Sports Exerc.

[CR62] Thum JS, Parsons G, Whittle T, Astorino TA (2017). High-intensity interval training elicits higher enjoyment than moderate intensity continuous exercise. PLoS ONE.

[CR63] Tokunaga A, Onda M, Okuda T, Teramoto T, Fujita I, Mizutani T, Kiyama T, Yoshiyuki T, Nishi K, Matsukura N (1995). Clinical significance of epidermal growth factor (EGF), EGF receptor, and c-erbB-2 in human gastric cancer. Cancer.

[CR99] Vieira VJ, Hu L, Valentine RJ, McAuley E, Evans EM, Baynard T, Woods JA (2009). Reduction in trunk fat predicts cardiovascular exercise training-related reductions in C-reactive protein. Brain Behav Immun.

[CR64] Vollaard NBJ, Metcalfe RS (2017). Research into the health benefits of sprint interval training should focus on protocols with fewer and shorter sprints. Sports Med.

[CR65] Vollaard NBJ, Metcalfe RS, Williams S (2017). Effect of number of sprints in an SIT session on change in VO_2max_: a meta-analysis. Med Sci Sports Exerc.

[CR66] Wagner PD, Olfert IM, Tang K, Breen EC (2006). Muscle-targeted deletion of VEGF and exercise capacity in mice. Respir Physiol Neurobiol.

[CR67] Wei T, Simko V (2017). R package “corrplot”: Visualization of a Correlation Matrix (Version 0.84). Available from https://github.com/taiyun/corrplot

[CR68] Weston KS, Wisløff U, Coombes JS (2014). High-intensity interval training in patients with lifestyle-induced cardiometabolic disease: a systematic review and meta-analysis. Br J Sports Med.

[CR69] Yasar Z, Dewhurst S, Hayes LD (2019). Peak power output is similarly recovered after three- and five-days' rest following sprint interval training in young and older adults. Sports.

[CR70] Yousefzadeh MJ, Schafer MJ, Noren Hooten N, Atkinson EJ, Evans MK, Baker DJ, Quarles EK, Robbins PD, Ladiges WC, LeBrasseur NK, Niedernhofer LJ (2018). Circulating levels of monocyte chemoattractant protein-1 as a potential measure of biological age in mice and frailty in humans. Aging Cell.

[CR71] Zanni F, Vescovini R, Biasini C, Fagnoni F, Zanlari L, Telera A, Di Pede P, Passeri G, Pedrazzoni M, Passeri M, Francheschi C, Sansoni P (2003). Marked increase with age of type 1 cytokines within memory and effector/cytotoxic CD8+ T cells in humans: a contribution to understand the relationship between inflammation and immunosenescence. Exp Gerontol.

[CR72] Żebrowska A, Jastrzębski D, Sadowska-Krępa E, Sikora M, Di Giulio C (2019). Comparison of the effectiveness of high-intensity interval training in hypoxia and normoxia in healthy male volunteers: a pilot study. Biomed Res Int.

[CR73] Zheng G, Qiu P, Xia R, Lin H, Ye B, Tao J, Chen L (2019). Effect of aerobic exercise on inflammatory markers in healthy middle-aged and older adults: a systematic review and meta-analysis of randomized controlled trials. Front Aging Neurosci.

